# Influenza Vaccination and Morbidity Among Sudanese Hajj Pilgrims During the 2025 Hajj

**DOI:** 10.3390/vaccines13111134

**Published:** 2025-11-03

**Authors:** Najim Z. Alshahrani, Mohammed R. Algethami, Abdulrahman M. Albeshry, Zuhier Awan, Wael AlZhrani, Osama A. Bugis, Abdullah Jaber Alsahafi, Harunor Rashid

**Affiliations:** 1 Department of Family and Community Medicine, College of Medicine, University of Jeddah, Jeddah 23218, Saudi Arabia; 2 College of Applied Medical Sciences, University of Jeddah, Jeddah 23218, Saudi Arabia; 3 Population Health Management, Jeddah First Health Cluster, Health Holding Company, Jeddah 21429, Saudi Arabia; 4 Children’s Hospital Westmead Clinical School, Sydney Infectious Diseases Institute, University of Sydney, Westmead, Sydney, NSW 2145, Australia; harunor.rashid@sydney.edu.au

**Keywords:** Hajj, healthcare utilization, morbidity, pilgrim, Sudan, vaccination, Saudi Arabia

## Abstract

Background: Little is known about morbidity patterns and healthcare utilization among specific Hajj pilgrim groups. This study examined influenza vaccination coverage, disease spectrum and healthcare utilization outcomes among Sudanese pilgrims during Hajj 2025. Methods: A cross-sectional analysis was conducted using de-identified patient records from the Saudi Health Electronic Surveillance Network for Sudanese Hajj pilgrims in 1–9 June 2025. Data included demographics, influenza vaccination status, healthcare utilization metrics, morbidities and temporal distribution of visits. Comparisons between hospital and primary healthcare center (PHC) attendees were performed using appropriate statistical tests. Results: A total of 1130 pilgrims sought care, with 88.6% (*n* = 1001) attending PHCs and 11.4% (*n* = 129) hospitals. Their mean age was 49.7 ± 12.9 years, and 67.9% (*n* = 767) were male. Influenza vaccination coverage was 79% (893/1130); vaccinated pilgrims had lower incidence of influenza-like illness (ILI) compared to unvaccinated pilgrims (5.2% vs. 15.2%, *p* < 0.01). Respiratory illnesses were the most frequent diagnoses (40.8% in PHCs and 24.8% in hospitals), followed by musculoskeletal disorders (24.5% and 16.3%, respectively). Compared to PHCs, presentation rate for chronic diseases was higher in hospitals (19.4% vs. 8.7%, *p* < 0.001), so was the median clinic time (14.1 vs. 8.6 min, *p* < 0.001). Healthcare utilization peaked on days 3–5 coinciding with the ‘Arafat Day’. Conclusions: Sudanese pilgrims most commonly presented with acute respiratory conditions, with PHCs managing the majority of cases, and influenza vaccination was protective against ILI. Findings emphasize the need for strong primary care, efficient resource allocation, and targeted preventive strategies to safeguard pilgrims’ health in the future.

## 1. Introduction

The annual Hajj pilgrimage represents one of the world’s largest recurrent mass gatherings, drawing millions of Muslims from over 180 countries to the holy cities of Saudi Arabia [1]. The number of pilgrims varies significantly each year, with recent estimates indicating over 1.67 million participants in the Hajj 2025 and a record high of more than 3.16 million in 2012 [2,3]. Prior to the COVID-19 pandemic, the combined total of international and local pilgrims frequently exceeded 2 million annually [4]. As one of the five pillars of Islam, Hajj holds profound spiritual significance, obligating all physically and financially capable practicing Muslims perform the pilgrimage at least once in their lifetime [5].

This dense concentration of individuals in a confined geographical area—coupled with physically demanding rituals and extreme environmental conditions—creates a unique context with significant public health implications [6]. Also, the shifting seasonal timing of Hajj, governed by the lunar calendar, introduces variability in climate and disease transmission dynamics, further complicating health preparedness and response efforts [7]. Consequently, morbidity and mortality among pilgrims remain a critical concern for health authorities, necessitating the deployment of robust and responsive health services to ensure pilgrim well-being. A substantial proportion of pilgrims present with pre-existing chronic diseases, with recent estimates indicating that annually over 300,000 may arrive with such comorbidities [8]. Cardiovascular diseases and diabetes are consistently reported as leading causes of death during Hajj. Previous studies indicate that cardiovascular conditions account for the majority of intensive care unit admissions and fatalities among pilgrims [9].

To meet the healthcare demands of Hajj, Mina hosts a comprehensive and integrated medical network that operates continuously throughout the event. In 2025, this network comprised 30 Primary Healthcare Centers (PHCs) and four hospitals, strategically distributed to optimize accessibility for pilgrims [10]. These facilities deliver a full spectrum of services, including urgent care, emergency stabilization, acute medical management, pharmacy, laboratory diagnostics, and imaging. The system is both dynamic and scalable; for instance, in 2022 there were 26 PHCs, with annual adjustments made to the number and scope of facilities in response to evolving public health needs and logistical challenges associated with each pilgrimage season [11].

The multinational nature of the Hajj gathering introduces additional complexities in disease epidemiology and health service delivery [12]. Pilgrims with compromised health status face increased susceptibility to infectious diseases, including respiratory infections such as influenza and Middle East respiratory syndrome coronavirus [13]. The risk profile of pilgrims is not uniform and is profoundly influenced by their country of origin. For instance, Sudan, as a key participant nation, presents a distinct epidemiological context that warrants specific investigation [14]. The country contends with a dual burden of disease: a high prevalence of endemic communicable diseases such as tuberculosis and malaria, alongside a rapidly growing incidence of non-communicable diseases including diabetes, hypertension, and cardiovascular disorders [15]. When Sudanese pilgrims embark on the Hajj, they carry this health background into the demanding pilgrimage environment, potentially increasing their vulnerability to adverse health outcomes and posing specific challenges for health service providers [16]. Also, Sudan being located in Sub-Saharan Africa within the ‘meningitis belt’, large intercontinental outbreaks of Hajj-associated meningococcal disease affect it, at times very severely [17].

Studies from African pilgrim cohorts have consistently highlighted the considerable health burden experienced during Hajj. A longitudinal study of South African pilgrims (n = 1138) reported that nearly two-thirds fell ill during the pilgrimage, with respiratory symptoms being the most prevalent [18]. Similarly, Egyptian surveillance identified laboratory-confirmed influenza in 14% of pilgrims, while vaccination uptake remained low [19]. Broader reviews of African and low- and middle-income country (LMIC) pilgrims confirm heightened vulnerability to respiratory infections and chronic disease exacerbations during Hajj, largely driven by crowding, heat exposure, and limited pre-travel preparedness [20]. Despite Sudan’s major representation at Hajj, few studies have characterized the morbidity profiles or healthcare-utilization trends of its pilgrims. The limited available reports primarily describe vaccination compliance and general preventive practices, with no detailed analysis of service performance or disease patterns among Sudanese attendees.

Each year, over 12,000 pilgrims from Sudan participate in the Hajj. Previous focused surveys have shown high compliance with mandatory vaccinations and preventive health measures, yet more than 60% of respondents reported one or more health symptoms during the pilgrimage. However, no study to date has systematically examined morbidity patterns or healthcare service utilization among this group.

This study leverages, for the first time, electronic health data from both primary care and hospital facilities at Hajj to assess healthcare utilization and morbidity patterns among Sudanese travelers who attended the pilgrimage in 2025. Unlike earlier descriptive surveys, this analysis integrates operational performance indicators—such as waiting times, examination duration, and total clinic stay—with clinical outcomes and vaccination status. The study also investigates the relationship between influenza vaccination and the occurrence of influenza-like illness (ILI). This study aims to add to the limited African Hajj health literature and offers practical insights for strengthening primary healthcare preparedness, vaccination strategies, and surveillance systems during future Hajj seasons. Specifically, the study seeks to describe the spectrum of diseases, temporal trends in healthcare visits, and key operational metrics such as waiting times and total clinic duration. Additionally, we hypothesize that influenza vaccination is associated with a lower occurrence of ILI among Sudanese pilgrims during Hajj.

## 2. Materials and Methods

### 2.1. Study Design and Setting

This cross-sectional study utilized de-identified patient records obtained directly from the electronic health record (EHR) systems of hospitals and PHCs in Mina, Makkah. The 2025 Hajj officially took place from the evening of Wednesday, 4 June, to Monday, 9 June. For the purposes of this study, we expanded the observation window to 1–9 June 2025, to capture encounters from pilgrims who arrived early or remained slightly beyond the ritual period, as well as to ensure inclusion of all relevant medical visits linked to the Hajj season. While the majority of pilgrims stay in Mina for 5–6 days in line with the main rituals, this extended timeframe reflects the operational realities of healthcare delivery during mass gatherings. Healthcare for pilgrims in Mina is delivered through temporary hospitals and PHCs, which are staffed by specialized medical teams, equipped with advanced resources, and supported by an ambulance fleet to ensure timely access to emergency care.

This study focused on Sudanese pilgrims, as Sudan constitutes one of the largest African contingents participating in Hajj. Understanding specific health needs of this group has been recognized as a national and regional public health priority. The Sudanese cohort was selected based on the completeness and reliability of their records within the Saudi Health Electronic Surveillance Network (HESN). The analysis was confined to the official Hajj operational period (1–9 June 2025), aligning with the timeframe when Mina facilities are fully operational and healthcare utilization by pilgrims typically peaks. This time-bound enhances comparability and reflects the actual demand for health services during Hajj.

### 2.2. Study Population and Data Sources

The study population consisted of all Sudanese pilgrims who sought medical care at the participating hospitals and PHCs during the specified study period. A total of 1130 patient encounters were included in the final analysis. Data were collected from EHR systems of the facilities, which routinely capture patient demographics, timing of service utilization, and clinical diagnoses for the duration of Hajj. Each record in the dataset represented a unique healthcare encounter documented in the HESN (Ministry of Health, Riyadh, Saudi Arabia) system. Accordingly, the unit of analysis in this study was the individual healthcare encounters (observations) rather than unique pilgrims. This approach was adopted because the EHR system registers each visit independently and does not include identifiers that link multiple visits by the same individual. Therefore, repeated visits by the same pilgrim, if any, were counted as separate encounters to accurately reflect overall healthcare utilization during the Hajj period.

All variables available in the dataset were included in the analysis to ensure comprehensive utilization of the available information. The selected indicators comprised facility type (hospital or PHC), visit-to-exam time, exam-to-discharge time, total clinic time, diagnosis category, and vaccination status. These variables represent the complete set of routinely recorded data in the HESN system during the Hajj period. They align with the World Health Organization (WHO) and the Saudi Ministry of Health recommendations for monitoring healthcare utilization and disease surveillance in mass gatherings. Their inclusion allows for a robust assessment of healthcare access, service efficiency, and morbidity trends among pilgrims, thereby supporting evidence-based public health planning and service optimization during Hajj.

### 2.3. Data Collection, Categorization, and Processing

Data were extracted from EHRs and included sociodemographic variables (age, gender), temporal information (day of Hajj on which care was sought), healthcare utilization indicators, and clinical information (final physician-assigned diagnosis). The healthcare utilization indicators were defined as follows: the visit-to-examination interval referred to the waiting time from patient registration until being called for medical examination; the examination-to-discharge interval represented the duration of the clinical consultation from the initial physician assessment until discharge from the facility; and the total clinic time reflected the overall time spent at the healthcare facility, measured from registration to discharge.

To enable structured analysis of morbidity patterns, all clinical diagnoses were systematically reviewed and classified into predefined categories based on clinical relevance. These categories comprised respiratory diseases, musculoskeletal/pain disorders, gastrointestinal conditions, dermatological conditions, chronic/metabolic diseases, urinary/renal conditions, injury/trauma, and general/miscellaneous presentations. Diagnoses that did not fit clearly within these categories were grouped as “Other.” Examples of conditions placed in the “Other” category included irritable bowel syndrome, allergy (unspecified), anemia, and headache.

Clinical diagnoses were classified into predefined morbidity categories through a two-step process. First, conditions were provisionally grouped according to key diagnostic terms (e.g., “fracture” under injury/trauma, “diabetes” under chronic/metabolic, “cough” under respiratory). Second, all classifications were reviewed by the study team to confirm accuracy and ensure consistency across cases. To ensure validity and reliability of the classification process, two independent physicians cross-verified the diagnostic groupings using standard ICD-10 terminology. Any discrepancy was resolved through discussion and consensus among the research team. This approach ensured that the diagnostic categories were both clinically accurate and reproducible.

Vaccination data were extracted from the HESN vaccination registry fields linked to each pilgrim record. Among the available variables, only influenza vaccination status was consistently and reliably recorded across all entries; therefore, it was included in the analysis. COVID-19 and other vaccination data—such as meningococcal or pneumococcal vaccines—were not uniformly captured in the dataset and could not be analyzed without introducing substantial bias. The dataset encompassed all available variables, including demographic (age, gender, age group), temporal (Hajj day number, visit and examination timestamps), clinical (diagnosis codes and categories), and operational (facility type, clinic name, visit-to-exam time, exam-to-discharge time, and total clinic time) fields. This comprehensive inclusion ensured that the analysis utilized the complete set of systematically recorded indicators available in HESN during the 2025 Hajj. A detailed summary of these variables, their definitions, and data sources is provided in Table S1.

Influenza-like illness (ILI) was defined in accordance with the World Health Organization (WHO) surveillance case definition: an acute respiratory infection characterized by a measured fever ≥ 38 °C and cough, with onset within the past 10 days [21]. In the present study, ILI was identified based on clinician-assigned final diagnoses recorded in the EHR that met this case definition. Laboratory confirmation of influenza virus infection was not required; thus, the analysis refers to “clinician-diagnosed ILI”, or “self-reported influenza.” This standardized definition aligns with both WHO and Saudi Ministry of Health surveillance protocols, facilitating comparability of findings from other mass-gathering health studies.

### 2.4. Statistical Analysis

Data were analyzed using R statistical software (version 4.4.1, R Foundation for Statistical Computing, Vienna, Austria). Descriptive statistics were presented as frequencies and proportions for categorical variables. Continuous variables were assessed for normality and described using means and standard deviations (±SD) or medians and interquartile ranges (IQRs), as appropriate. Associations between categorical variables (e.g., facility type and gender) were assessed using the Chi-square test. Differences in continuous variables (e.g., wait times) between hospitals and PHCs were analyzed using the independent *t*-test for normally distributed data or the Mann–Whitney U test for non-parametric data. The association between influenza vaccination status and influenza-like illness (ILI) was also analyzed using the Chi-square test. In this study, ILI diagnoses were made by attending physicians in the healthcare facilities, based on the World Health Organization (WHO) operational definition of an acute respiratory illness with measured fever ≥ 38 °C and cough, with onset within the last 10 days. A two-sided *p*-value of <0.05 was considered statistically significant.

## 3. Results

### 3.1. Sociodemographic Characteristics

A total of 1130 unique Sudanese pilgrims attended healthcare facilities during the 2025 Hajj, with the majority presenting to PHCs (n = 1001, 88.6%) compared to hospitals (n = 129, 11.4%) (Table 1). Males represented 67.9% (n = 767) of the cohort, with the remaining 32.1% (n = 363) being females; the gender distribution did not significantly differ between hospitals and PHCs (*p* = 0.37). The mean age of pilgrims was 49.7 ± 12.9 years, with no significant difference between those attending hospitals (51.0 ± 14.2 years) and PHCs (49.6 ± 12.7 years). Half of the pilgrims (50.6%; n = 572) were aged 40–59 years, 25.3% (n = 286) were ≥60 years, and 24.1% (n = 272) were younger than 40 years. Attendance by age group showed a trend toward older pilgrims utilizing hospitals slightly more than PHCs, with the highest hospital attendance among those ≥60 years (13.6%). Pilgrim visits were distributed across the Hajj days, with the largest proportions on Day 3 (26.2%), Day 5 (25.6%), and Day 4 (23.3%). When compared with PHCs, hospital utilization varied significantly by Hajj day, peaking on Day 4 (15.6% of hospital visits cf. 84.4% of PHC visits, *p* = 0.03).

### 3.2. Service Utilization Metrics

Overall, pilgrims experienced a median visit-to-exam time of 5.67 min (IQR: 3.0–9.8), with hospitals requiring slightly longer times than PHCs (7.33 vs. 5.27 min, *p* < 0.001) (Table 2). Similarly, the median exam-to-discharge time was longer in hospitals compared to PHCs (4.10 min, IQR: 2.5–6.8 vs. 2.27 min, IQR: 1.5–4.2; *p* < 0.001), resulting in a longer median total clinic time in hospitals than PHCs (14.10 vs. 8.60 min, *p* < 0.001). The majority of patients were evaluated promptly: 83.9% within 15 min (82.2% in hospitals vs. 84.1% in PHCs, *p* = 0.09), 11.8% within 30 min (13.2% vs. 11.6%), 2.2% within 60 min (2.3% vs. 2.2%), and 2.1% waited longer than 60 min (2.3% vs. 2.1%).

### 3.3. Distribution of Disease Categories

**Figure 1:**
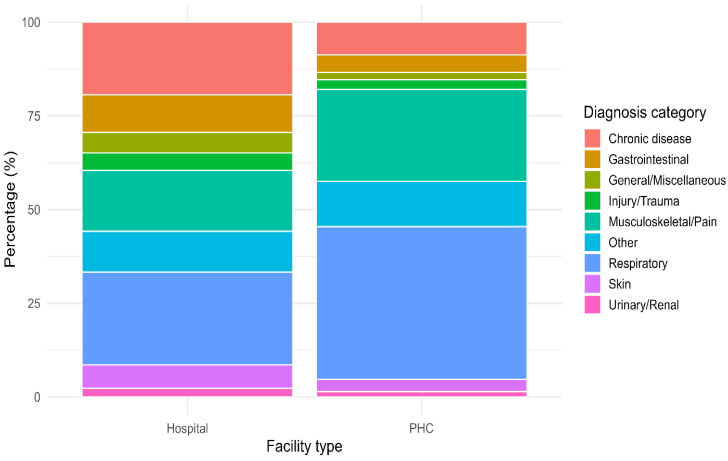
Distribution of diseases categories among Sudanese pilgrims during the 2025 Hajj, stratified by facility type.

Figure 1 illustrates the distribution of morbidity categories among Sudanese pilgrims, stratified by facility type. Respiratory conditions were the most frequently reported diagnoses, accounting for 24.8% of cases in hospitals and 40.8% in PHCs. Musculoskeletal/pain disorders were also common, representing 16.3% of hospital cases and 24.5% of PHC cases. Chronic diseases were more prevalent in hospitals than in PHCs (19.4% vs. 8.7%, *p* < 0.001), whereas gastrointestinal complaints were relatively less common in both settings (10.1% in hospitals and 4.7% in PHC). Other notable findings include injuries/trauma (4.7% in hospitals, 2.6% in PHC), skin conditions (6.2% in hospitals, 3.3% in PHC), and urinary/renal problems (2.3% in hospitals, 1.4% in PHC).

Among respiratory-related conditions, the most frequent diagnoses were acute nasopharyngitis (common cold, 32.7%), acute upper respiratory infection, unspecified (18.6%), and acute pharyngitis (18.2%), which together accounted for nearly 70% of all cases (Table 3). Other notable conditions included acute tonsillitis (unspecified 8.0%; specified 3.0%), cough (7.3%), and acute sinusitis (3.4%). Less frequent presentations such as conjunctivitis (1.4%), unspecified asthma (0.9%), and various rare respiratory and ear–nose–throat disorders were grouped under other diagnoses (3.4%).

### 3.4. Influenza Vaccination and Influenza-like Illness

Of the 1130 Sudanese pilgrim encounters included, 893 (79.0%) were vaccinated against influenza while the rest (21.0%) were not (Table 4). Overall, self-reported influenza or ILI was observed in 7.3% of the cohort. The occurrence of ILI was significantly lower among vaccinated compared to unvaccinated pilgrims (5.2% vs. 15.2%, *p* < 0.001), demonstrating a statistically significant protective effect of influenza vaccination against self-reported influenza during the 2025 Hajj.

### 3.5. Temporal Distribution of Visits

**Figure 2:**
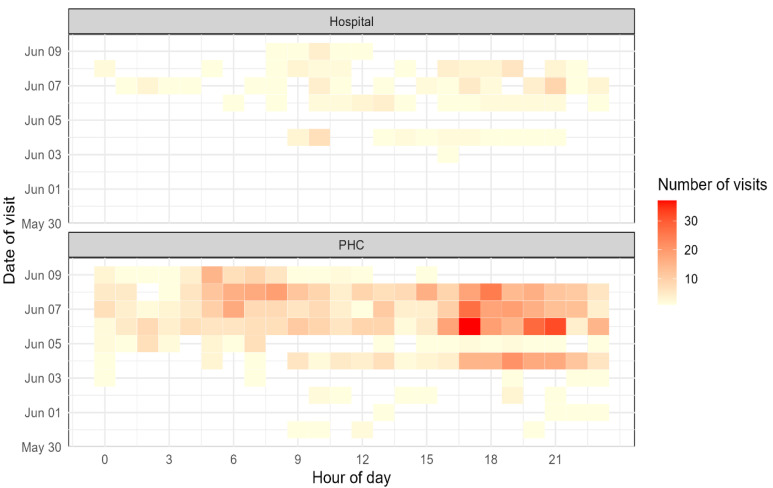
Temporal distribution of pilgrim visits to healthcare facilities during the 2025 Hajj.

Figure 2 illustrates the temporal distribution of healthcare visits by Sudanese pilgrims across hospitals and PHCs. Visits to PHCs were more frequent and widely distributed across the Hajj period, with clear peaks in the mornings (06:00–10:00) and evenings (16:00–21:00), particularly on 5–7 June, when activity reached its peak (>30 visits per hour). In contrast, hospital visits were fewer and more sporadic, clustering mainly in the late afternoons and evenings, with moderate peaks observed on 4–6 June. A noticeable dip in visits occurred on 5 June for both hospitals and PHCs.

### 3.6. Temporal Trends of Disease Categories

**Figure 3:**
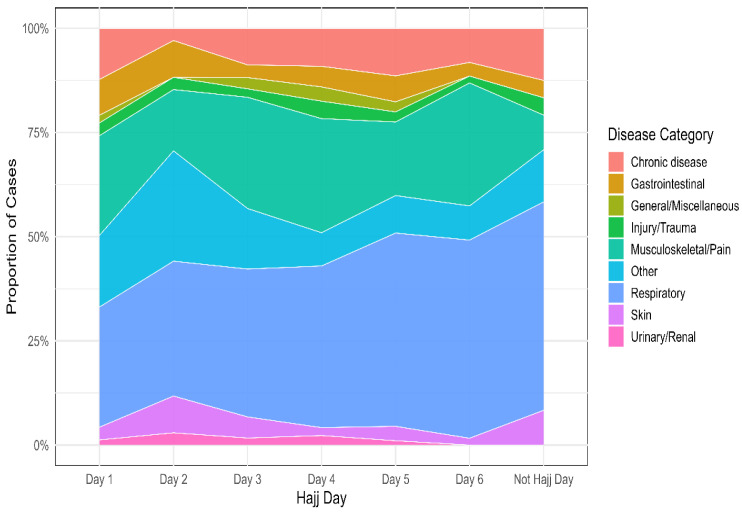
Temporal trends of healthcare utilization among Sudanese pilgrims during the 2025 Hajj.

Figure 3 illustrates the daily distribution of morbidity categories across the six Hajj days. Respiratory conditions were the most prevalent, gradually increasing over the days and peaking on Day 6 (47.5%), followed by musculoskeletal and pain-related disorders, which consistently contributed a substantial proportion of cases (14.7–29.5%) reaching their peaks on Day 6. The “Other” category, which mainly comprised headache and allergy cases, fluctuated across the period, with the highest proportion observed on Day 2 (26.5%). Visits for chronic diseases accounted for 2.9–12% of daily cases, with a notable dip on Day 2 (2.9%). Gastrointestinal conditions represented 3.0–8.8% of cases, while injury/trauma, skin, general/miscellaneous, and urinary/renal conditions were less frequent, each generally comprising less than 5% of daily cases.

## 4. Discussion

This cross-sectional study provides one of the first focused analyses of morbidity patterns and healthcare utilization among Sudanese pilgrims during the 2025 Hajj. The analysis was based on de-identified electronic health records from hospitals and PHCs in Mina, Makkah, which exclusively serve pilgrims during the Hajj season. The dataset included all Sudanese pilgrims who sought medical care at these facilities during the study period, ensuring comprehensive coverage of this population. Our findings reveal a disease profile dominated by respiratory illnesses, musculoskeletal complaints, and chronic disease exacerbations, with patterns of healthcare utilization reflecting both the acute demands of mass gatherings and the organizational structure of Hajj health services.

Respiratory illnesses emerged as the leading cause of healthcare visits, accounting for nearly one-third of all diagnoses. This finding aligns with decades of Hajj literature, where upper respiratory tract infections consistently dominate morbidity [22,23,24]. In our cohort, common cold, pharyngitis, and unspecified upper respiratory infections comprised almost 70% of respiratory diagnoses, a distribution remarkably similar to that reported among South African pilgrims in 2023, where sore throat, cough, and nasal congestion predominated [24]. This convergence underscores the near-universal vulnerability of pilgrims to respiratory pathogens, driven by crowd density, shared accommodations, and physically demanding rituals [25].

Interestingly, presentations for chronic diseases accounted for a disproportionately higher burden in hospitals (19.4%) compared to PHCs (8.7%). This pattern suggests that pilgrims with comorbidities may preferentially seek, or be referred to, higher-level facilities when symptoms exacerbate. A similar trend in the high prevalence of chronic illnesses among pilgrims was reported by Mahomed et al. (2024), who found that nearly half of South African pilgrims had pre-existing chronic conditions, most commonly hypertension and diabetes, which predisposed them to illness during and after Hajj [24]. Our findings thus emphasize the importance of stratifying health services, ensuring that hospitals are adequately prepared for pilgrims requiring specialized or emergency care, while PHCs continue to handle the bulk of self-limiting acute illnesses [26].

The distribution of other conditions, including musculoskeletal complaints, gastrointestinal illness, trauma, and dermatological conditions, mirrors the pattern observed in previous Hajj cohorts [23,27]. Musculoskeletal pain, often related to prolonged walking, standing, ritual movements, and muscle strain or cramps, consistently ranks among the top three diagnoses, reflecting the physical strain of Hajj rituals [28]. Dehydration, common in hot and crowded environments, may further contribute to the occurrence of muscle cramps. Gastrointestinal and skin conditions were less frequent, but their presence highlights the contribution of environmental stressors such as heat, dehydration, and crowding [29]. Although less commonly reported, headache was frequently documented in clinical records and may plausibly be linked with dehydration and heat exposure—both well-established risk factors during Hajj. This underscores the importance of promoting adequate hydration and ensuring access to cooling facilities as integral components of preventive health measures for pilgrims.

In our study cohort, we observed a marked preference for PHCs, which handled nearly nine out of ten patient encounters. This reflects the intentional design of the Hajj healthcare system, where PHCs serve as the first point of contact and hospitals absorb more complex cases [30]. The slightly longer median consultation and discharge times in hospitals compared to PHCs were expected, given the higher clinical complexity of cases managed in hospitals [30]. Importantly, the majority of pilgrims were seen within 15 min of arrival, reflecting high efficiency of service delivery. Comparable efficiency has been noted in earlier Hajj studies, such as Yezli et al. (2022), who reported an average dispensing time of eight minutes for prescribed medications in PHCs, suggesting that streamlined systems remain a cornerstone of Hajj healthcare preparedness [23].

Temporal analysis revealed that healthcare demand peaked on Days 3–5 of Hajj, corresponding to the busiest ritual days, particularly the ‘Arafat Day’ when all pilgrims assemble at the foot of Mount Arafat. This surge is consistent with earlier work showing increased utilization around and after Arafat [31]. However, a brief decline in both hospital and PHC visits was noted on 5 June, coinciding with the key pilgrim rites at Arafat and subsequent mass mobilization to Muzdalifah. During this period, the majority of pilgrims remain outside Mina, where the studied facilities are situated, leading to a temporary reduction in service utilization. The clustering of hospital visits in the late afternoon and evening may be explained by pilgrims delaying presentation until rituals were completed, while PHC visits were more evenly distributed across the day. These temporal patterns provide important operational insights for staffing allocation, suggesting that PHCs require maximal staffing during morning and evening peaks, while hospitals must prepare for surges in the late afternoon. In addition, given the critical role of PHCs in delivering first-line care—particularly in Arafat where patient demand intensifies—strategic expansion of PHC capacity in these zones should be considered to better accommodate peak patient loads [32].

The daily distribution of disease categories further reflects the cumulative burden of exposure and fatigue. Respiratory conditions steadily increased throughout the Hajj period, peaking on the final day, consistent with the incubation period of viral infections and ongoing transmission in crowded settings [3]. Musculoskeletal complaints also surged toward the end of Hajj, likely reflecting physical exhaustion from sustained rituals [33]. By contrast, gastrointestinal conditions and trauma were sporadic and less temporally patterned. The low frequency of gastrointestinal illness may reflect the effectiveness of strict food safety standards and continuous monitoring during Hajj, which help reduce risks of foodborne outbreaks [34,35].

A particularly noteworthy finding was the likely protective effect of influenza vaccination, with vaccinated pilgrims showing a three-fold lower risk of ILI compared to unvaccinated pilgrims. This result echoes evidence from both observational studies and systematic reviews [36,37], demonstrating that while flu vaccination does not totally eliminate infection risk among mass gathering attendees, it does substantially reduce disease burden and clinical severity of infection [38]. The seemingly positive finding reinforces the call for annual updates to vaccine formulations and for timely administration of influenza vaccines in pilgrims’ home countries prior to travel.

In our cohort, vaccination uptake was decent (79%), suggesting strong compliance with public health recommendations, but the persistence of ILI cases among vaccinated pilgrims indicates potential mismatches with circulating strains or waning immunity, as noted in earlier studies [22]. The findings of this study highlight the predictable health dynamics associated with the Hajj pilgrimage. Musculoskeletal complaints are largely attributable to the intense physical exertion required during rituals, while respiratory infections reflect the close contact and crowd density inherent to mass gatherings. These patterns are consistent with previous Hajj studies and underscore the unique challenges posed by such an extraordinary event.

The 2025 season benefited from substantial advances in infrastructure and public health measures, including shaded areas, hydration and cooling stations, green and sustainable facilities, AI-assisted crowd management, and drone-supported emergency response. Nevertheless, the persistence of common conditions such as respiratory and musculoskeletal disorders indicates that these are intrinsic risks rather than system deficiencies [39,40].

To further mitigate these challenges, emphasis should be placed on pre-departure education for pilgrims, particularly by Hajj campaign organizers, covering preventive practices, vaccination, hydration, and safe physical preparation. Strengthening such upstream measures, alongside existing innovations in surveillance and facility readiness, will help ensure safer and more sustainable pilgrimages, especially for older adults and those with chronic illnesses.

This study has several limitations that should be considered when interpreting the findings. First, the analysis was based on routinely collected health records from pilgrims who attended healthcare facilities, which may underestimate the true burden of illness, as individuals with mild or self-limiting conditions may not have sought medical attention. The dataset therefore represents a service-based sample rather than a population-based one, capturing only individuals who presented to hospitals or PHCs during the study period. Consequently, the findings reflect healthcare utilization patterns among facility-attending pilgrims rather than the full spectrum of morbidity experienced by the broader Sudanese pilgrim population. Second, clinical diagnoses were derived from routine physician assessments and were not consistently confirmed with laboratory or imaging investigations, introducing the possibility of diagnostic misclassification, particularly for respiratory illnesses. Third, the study population was limited to Sudanese pilgrims, which may restrict the generalizability of the findings to pilgrims of other nationalities who may differ in health status, risk factors, and vaccination coverage.

Fourth, although regression models were initially constructed to identify predictors of ILI and healthcare utilization, the limited number of covariates and high within-group homogeneity yielded nonsignificant results with minimal interpretive value. As a result, the analysis was restricted to descriptive and bivariate approaches. We recommend that future studies utilize larger and more granular datasets to enable robust multivariable regression modeling and more comprehensive explorations of these associations. Finally, the cross-sectional design provides a snapshot of morbidity and healthcare utilization during the 2025 Hajj but does not allow assessment of longitudinal outcomes, such as disease progression, complications, or post-Hajj health status.

## 5. Conclusions

This study provides valuable insights into the morbidity patterns and healthcare utilization of Sudanese pilgrims during the 2025 Hajj. Respiratory illnesses and musculoskeletal disorders were the predominant health problems, with PHCs managing the vast majority of encounters, while hospitals primarily handled chronic disease exacerbations and complex conditions. The temporal clustering of visits around the Day of Arafat and the observed protective association of influenza vaccination highlight key considerations for preventive health strategies and service planning. Given that most health needs during Hajj are managed at the primary care level, strengthening PHC preparedness should be a central priority in future health system planning. Key strategies include ensuring adequate staffing during peak periods, optimizing triage and referral systems, and enhancing capacity to manage common acute minor illnesses. Hospitals should continue to function as referral centers for severe or specialized cases but should not be the primary focus of surge preparedness efforts. Overall, these findings underscore the importance of sustained investment in vaccination programs, preventive education, and the operational readiness of PHCs to ensure safe, efficient, and equitable healthcare delivery during future Hajj seasons.

## Figures and Tables

**Table 1:** Sociodemographic characteristics of Sudanese pilgrims attending healthcare facilities during Hajj 2025, stratified by facility type (Hospital vs. PHC).

Variable		Total (*n* = 1130) *N* (Column%)	Hospitals (*n* = 129) *N* (Row%)	PHCs (*n* = 1001) *N* (Row%)	*p*-Value ^1^
Gender	Female	363 (32.1%)	37 (10.2%)	326 (89.8%)	0.370
	Male	767 (67.9%)	92 (12.0%)	675 (88.0%)	
Age group	<40 years	272 (24.1%)	31 (11.4%)	241 (88.6%)	0.050
	40–59 years	572 (50.6%)	59 (10.3%)	513 (89.7%)	
	≥60 years	286 (25.3%)	39 (13.6%)	247 (86.4%)	
	Mean age (±SD)	49.7 (12.9)	51.0 (14.2)	49.6 (12.7)	
Hajj days	Before June 4 (Not Hajj Day)	24 (2.1%)	1 (4.2%)	23 (95.8%)	0.030 *
	Day 1—Wed, 4 June	163 (14.4%)	22 (13.5%)	141 (86.5%)	
	Day 2—Thu, 5 June	34 (3.0%)	0 (0.0%)	34 (100.0%)	
	Day 3—Fri, 6 June	296 (26.2%)	25 (8.4%)	271 (91.6%)	
	Day 4—Sat, 7 June	263 (23.3%)	41 (15.6%)	222 (84.4%)	
	Day 5—Sun, 8 June	289 (25.6%)	32 (11.1%)	257 (88.9%)	
	Day 6—Mon, 9 June	61 (5.4%)	8 (13.1%)	53 (86.9%)	

^1^ *p*-values represent differences between hospitals and PHCs. Categorical variables (gender, age group, Hajj days) were compared using the chi-square test, while the mean age (continuous variable) was compared using an independent *t*-test. * Statistically significant *p*-value < 0.05.

**Table 2:** Service utilization metrics of Sudanese pilgrims during Hajj 2025 by facility type (Hospital vs. PHC).

Variable		Total (*n* = 1130) *N* (Column%)	Hospitals (*n* = 129) *N* (Row%)	PHCs (*n* = 1001) *N* (Row%)	*p*-Value
Median visit-to-exam time (minutes, [IQR])		5.67 (3.0–9.8)	7.33 (5.0–12.5)	5.27 (3.0–9.0)	<0.001 *
Median exam-to-discharge time (minutes, [IQR])		2.42 (1.5–4.5)	4.10 (2.5–6.8)	2.27 (1.5–4.2)	<0.001 *
Median total clinic time (minutes, [IQR])		9.17 (6.5–15.0)	14.10 (9.0–20.0)	8.60 (6.0–13.5)	<0.001 *
Percent of time taken to examine the patients	% seen within 15 min	948 (83.9%)	106 (82.2%)	842 (84.1%)	0.090
	% seen within 30 min	133 (11.8%)	17 (13.2%)	116 (11.6%)	
	% seen within 60 min	25 (2.2%)	3 (2.3%)	22 (2.2%)	
	% seen more than 60 min	24 (2.1%)	3 (2.3%)	21 (2.1%)	

* Statistically significant *p*-value < 0.05.

**Table 3:** Distribution of final respiratory-related diagnoses among symptomatic Sudanese pilgrims during the 2025 Hajj (*N* = 440).

Final Diagnosis	*n* (%)
Acute nasopharyngitis (common cold)	144 (32.7)
Acute upper respiratory infection, unspecified	82 (18.6)
Acute pharyngitis	80 (18.2)
Acute tonsillitis, unspecified	35 (8.0)
Cough	32 (7.3)
Acute sinusitis	15 (3.4)
Acute tonsillitis	13 (3.0)
Conjunctivitis	6 (1.4)
Acute pharyngitis, unspecified	4 (0.9)
Asthma, unspecified	4 (0.9)
Disease of upper respiratory tract, unspecified	4 (0.9)
Acute atopic conjunctivitis	3 (0.7)
Acute upper respiratory infections of multiple/unspecified sites	3 (0.7)
Other diagnoses *	15 (3.4)

* Includes chronic sinusitis, asthma, acute laryngopharyngitis, allergic rhinitis due to pollen, bronchopneumonia unspecified, and others.

**Table 4:** Association between influenza vaccination status and occurrence of ILI among Sudanese pilgrims during the 2025 Hajj.

Influenza Vaccination	Total *N* (Column %)	No ILI *N* (Row%)	ILI *N* (Row%)	*p*-Value
Not vaccinated	237 (21.0)	201 (84.8)	36 (15.2)	<0.001 ^1^
Vaccinated	893 (79.0)	847 (94.8)	46 (5.2)	
Total	1130 (100)	1048 (92.7)	82 (7.3)	

^1^ Statistically significant *p*-value < 0.05. ILI: Influenza-like illness.

## Data Availability

The data used in this study were obtained from the Saudi Health Electronic Surveillance Network (HESN), Ministry of Health, Riyadh, Saudi Arabia. Due to privacy and ethical restrictions imposed by the Ministry of Health, the data are not publicly available. De-identified data may be made available from the corresponding author upon reasonable request and with permission from the Ministry of Health.

## Supplementary Materials

The following supporting information can be downloaded at: https://www.mdpi.com/article/10.3390/vaccines13111134/s1, Table S1: Variables and data sources used in the analysis.
