# Supplementary material for: Influenza Vaccination and Morbidity Among Sudanese Hajj Pilgrims During the 2025 Hajj
Source: Vaccines (Basel). 2025 Nov 3;13(11):1134. doi: 10.3390/vaccines13111134 (PMC12656401; doi:10.3390/vaccines13111134)
Supplement: Supplementary file 1 [file vaccines-13-01134-s001.zip › vaccines-3925417-supplementary.pdf]

Table S1. Variables and data sources

Variable Name	Definition / Description	Type	Source / Data Field	Notes
**Facility_type**	Type of healthcare facility where the patient was treated PHC or Hospital).	Categorical	HESN facility registry	Used to compare healthcare utilization between settings.
**Facility_name**	Specific name or identifier of the health facility.	Text	HESN facility metadata	Used for facility-level descriptive summaries.
**Gender**	Patient's gender (0 = male, 1 = female).	Categorical	EHR patient demographic record	—
**Age**	Patient's age in years at the time of visit.	Continuous	EHR patient demographic record	Used to calculate age group.
**Age_group**	Categorized age groups (<40 years, 40–59 years, ≥60 years).	Categorical	Derived from age variable	Used for stratified analyses.
**CLINIC**	Type of clinic or service attended within	Text	EHR clinical encounter field	—

	the facility (e.g., General Clinic).			
**Hajj_Day_number**	Sequential day of the Hajj period on which the visit occurred (Day 1–9).	Ordinal	Derived from visit date	Used for temporal trend analysis.
**VISIT_DATE**	Date and time of patient registration at the facility.	Timestamp	EHR system log	Used to compute visit intervals.
**EXAMINED_DATE**	Date and time when the patient was examined by a physician.	Timestamp	EHR system log	—
**visit-to-exam time**	Interval (in minutes) from registration to start of examination.	Continuous	Computed from VISIT_DATE and EXAMINED_DATE	Indicator of waiting time and access efficiency.
**seen_within_minutes**	Categorical version of visit-to-exam time (≤15, 16–30, 31–60, >60 minutes).	Categorical	Derived variable	Used for service timeliness reporting.

**exam-to-discharge time**	Interval (in minutes) between start of examination and discharge.	Continuous	Computed from EXAMINED_DATE and Discharge_Date	Indicator of consultation duration.
**total_clinic_time**	Total time (in minutes) spent in the facility, from registration to discharge.	Continuous	Derived from visit and discharge timestamps	Reflects overall service time.
**FINAL_DIAGNOSIS_CODE**	ICD-10 code assigned by the attending physician.	Text	EHR diagnostic field	Standardized for morbidity classification.
**FINAL_DIAGNOSIS**	Description of final clinical diagnosis.	Text	EHR diagnostic field	Used to categorize morbidity types.
**Diagnosis_category**	Grouped clinical category (e.g., Respiratory, Gastrointestinal, Injury/Trauma, Other).	Categorical	Derived variable	Created for morbidity pattern analysis.
**DISCHARGE_STATUS**	Patient's disposition after visit (e.g.,	Categorical	EHR encounter summary	—

	Discharged from clinic, Referred).			
**Influenza_vaccine**	Indicator of receipt of influenza vaccination for the 2025 season (1 = vaccinated, 0 = not vaccinated).	Binary	HESN vaccination registry	Only vaccination variable consistently recorded across records.
**Influenza-like illness (ILI)**	Clinical diagnosis consistent with WHO definition: acute respiratory illness with measured fever ≥38°C and cough, onset within the last 10 days.	Binary	Derived from FINAL_DIAGNOSIS	Used to assess vaccination effectiveness.
**Discharge_Date**	Date and time of discharge from facility.	Timestamp	EHR system log	Used for calculation of time intervals.

**Category**	Simplified or duplicate diagnostic grouping field used for internal validation.	Text	EHR export	Not used in statistical analysis.

HESN: Saudi Health Electronic Surveillance Network, EHR: Electronic health record, PHC: Primary Healthcare Center.

Data Sources: All data were obtained from the Saudi Health Electronic Surveillance Network (HESN) as part of routine clinical documentation during the 2025 Hajj. No personally identifiable information was accessed or included in the analysis. Variables were included as recorded; no fields were excluded or imputed.
